# Posttraumatic Scrotal Reconstruction with a Pedicled “Extended” Superficial Circumflex Iliac Artery Perforator Flap: A Case Report

**DOI:** 10.1055/a-2166-8783

**Published:** 2024-05-09

**Authors:** Lucía Sisternas Hernández, Susana López Fernández, Paúl D. Zamora Alarcón, Carmen Vega García, Laura Torrano Romero, Manuel Fernández Garrido

**Affiliations:** 1 Department of Plastic and Reconstructive Surgery, Hospital de la Santa Creu i Sant Pau, Barcelona, Spain

**Keywords:** penoscrotal reconstruction, SCIP flap, pedicled flap

## Abstract

The superficial circumflex iliac artery (SCIA) perforator (SCIP) flap has been used for scrotal reconstruction after Fournier's gangrene, skin cancer, or infections. However, there are few publications with regard to penoscrotal reconstruction after a traumatic injury with this flap. In this article, we propose a new SCIP flap variation, the “extended” or “direct” SCIP flap, to effectively reconstruct a wide scrotal defect after a traumatic injury. The “extended” SCIP flap is designed medial and cranial to the anterosuperior iliac spine (ASIS) using the superficial branch of the SCIA as the main pedicle.

## Introduction

Penoscrotal defects can arise due to various causes, typically occurring subsequent to Fournier's gangrene, skin malignancies, infections, or traumatic incidents. 1 2 3 Throughout the traditional approach to penoscrotal reconstruction, a variety of techniques have been employed, such as skin grafts, local flaps (including fasciocutaneous or musculocutaneous flaps), tissue expanders, and free flaps. 4 5 6 7 8 However, it is important to note that the use of skin grafts can often result in skin contracture. 9 Conversely, the use of thick flaps, such as traditional local and free flaps, poses challenges in terms of insertion and frequently yields suboptimal aesthetic outcomes due to their bulky nature, which can give rise to additional complications.

To address these limitations, there is a need for a thin yet reliable flap in the realm of scrotal and penile reconstruction. Among the various options available, the superficial circumflex iliac artery perforator (SCIP) flap seems a promising choice, fulfilling the aforementioned criteria. 10 11

Notably, there have been documented cases in which an SCIP propeller flap has been successfully utilized for scrotal reconstruction following conditions like Fournier's gangrene, extramammary Paget's disease (a type of skin cancer), or infections resulting from foreign-body injections. 1 12 The etiology of the defect does not represent a big difference for the choice of the reconstructive technique, but proper functional and cosmetic reconstruction of the penoscrotal tissue is especially important in posttraumatic injuries as they usually happen in younger patients. 13

The purpose of this article is to propose a new SCIP flap variation, the “extended” or “direct” SCIP flap, 14 to effectively reconstruct a wide scrotal defect after a traumatic injury.

## Case

**Fig. 1:**
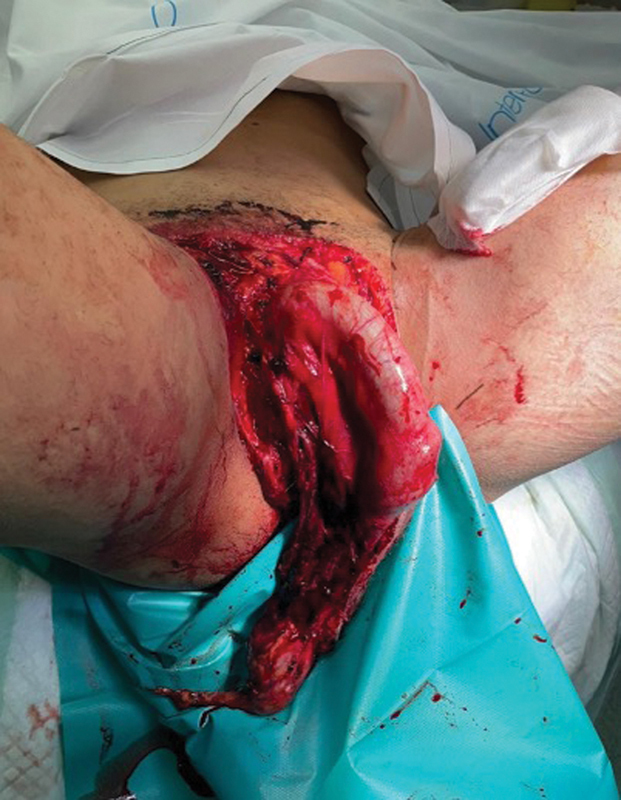
Avulsed scrotum after traumatic injury.

**Fig. 2:**
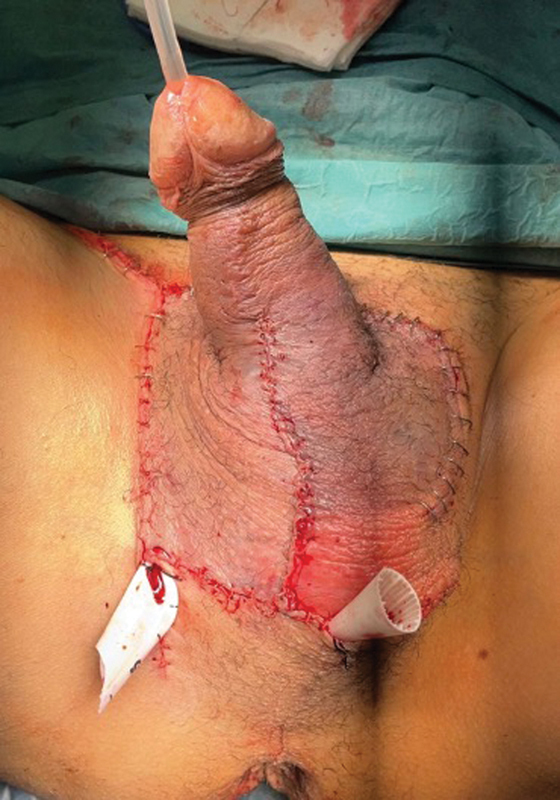
Postoperative image after relocation of the avulsed skin flaps.

**Fig. 3:**
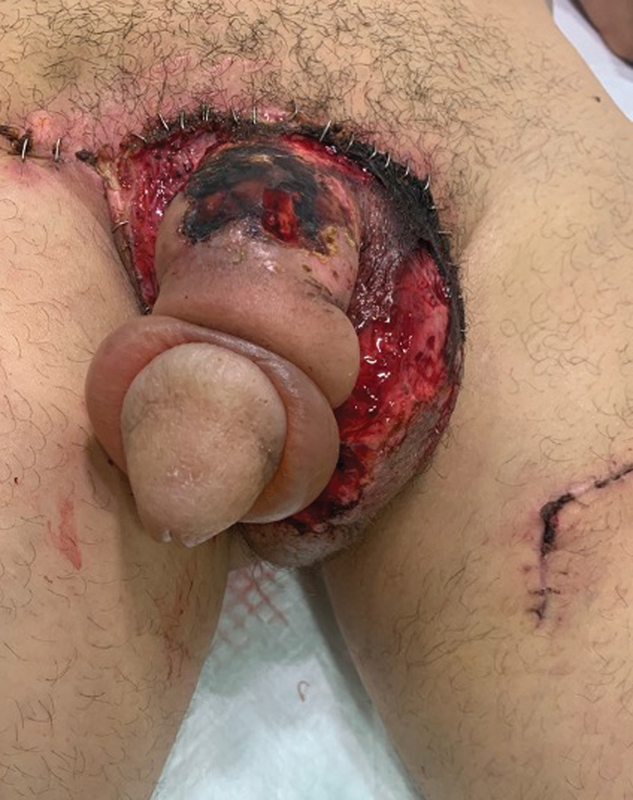
Flap necrosis.

A male patient, aged 55, was admitted to our medical facility due to wide avulsion of the scrotal skin after a traumatic injury in a bicycle accident ( Fig. 1 ). He underwent an emergency surgery with right orchiectomy and relocation of the avulsed scrotum skin flap ( Fig. 2 ). Nevertheless, some days after, he developed scrotal flap necrosis ( Fig. 3 ) that required a wide debridement. It ended up with a 12 × 8 cm (96 cm ^2^ ) soft tissue defect over the right scrotum and proximal penis dorsum.

In order to address the resulting defect, we proposed employing the “extended” or “direct” superficial circumflex iliac artery (SCIA) SCIP flap. This particular modification of the SCIP flap focuses exclusively on the superficial branch of the SCIA, with the skin island being designed in a medial and cranial position relative to the anterosuperior iliac spine (ASIS). 14

To assist in the preoperative planning, we used Angio-CT and Doppler sonography to precisely identify the point where the superficial branch of the SCIA intersects with the Hesselbach's fascia. Based on our department angiographic study, 14 this point of exit is typically situated within a circumference of approximately 21 mm radius, positioned 18 mm medially and 17 mm distally from the ASIS in approximately 90% of patients.

**Fig. 4:**
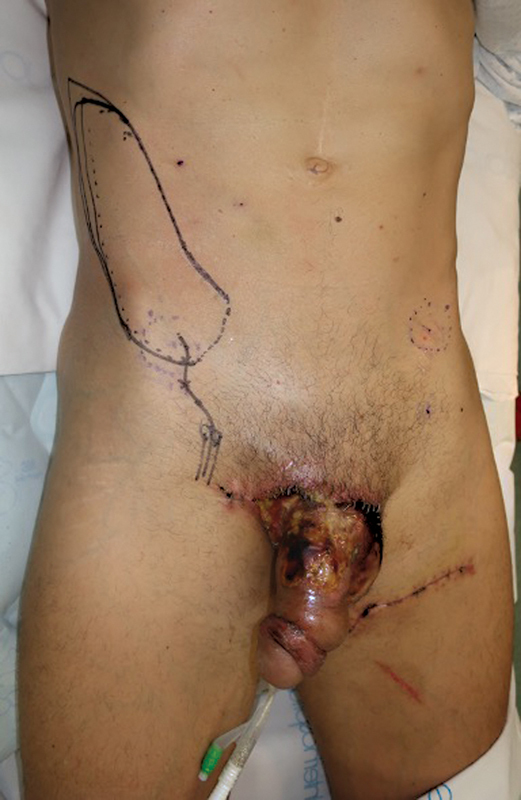
“Direct” SCIP design, medial and cranial to the ASIS. ASIS, anterosuperior iliac spine; SCIP, superficial circumflex iliac artery perforator.

**Fig. 5:**
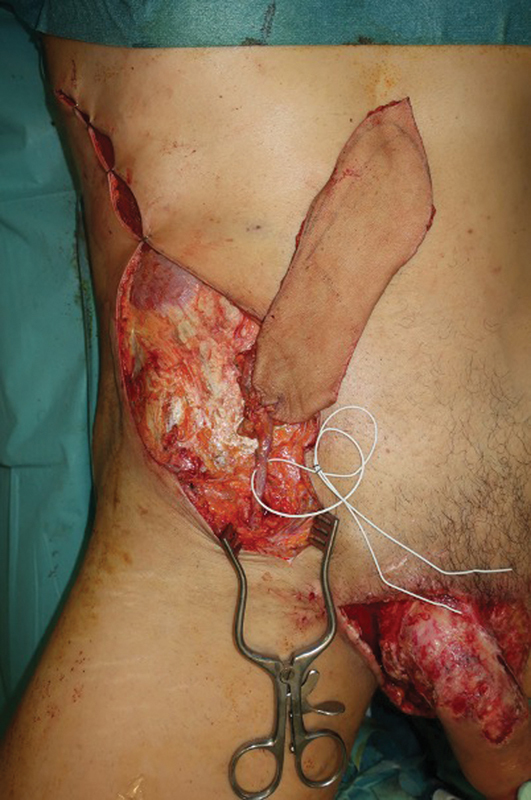
SCIP flap harvested based on the superficial branch of the SCIA dissected until its origin in the SCIA. SCIA, superficial circumflex iliac artery; SCIP, superficial circumflex iliac artery perforator.

**Fig. 6:**
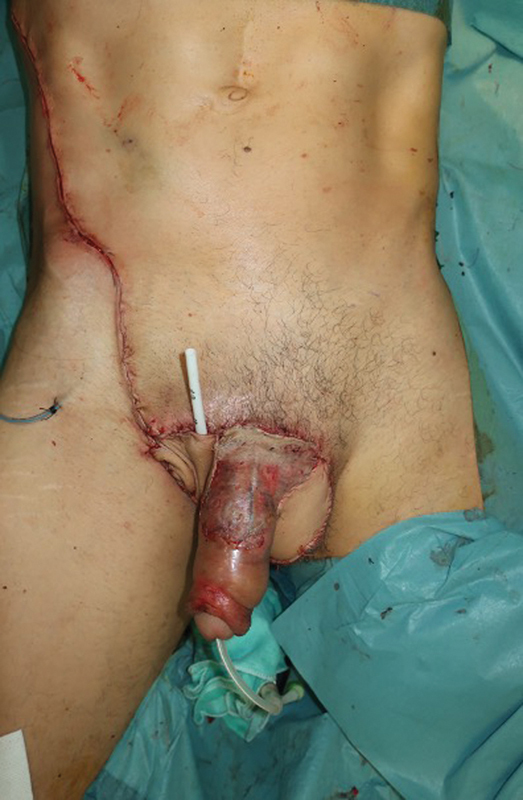
Immediate postoperative image after reconstruction of the penoscrotal defect with the SCIP flap. SCIP, superficial circumflex iliac artery perforator.

**Fig. 7:**
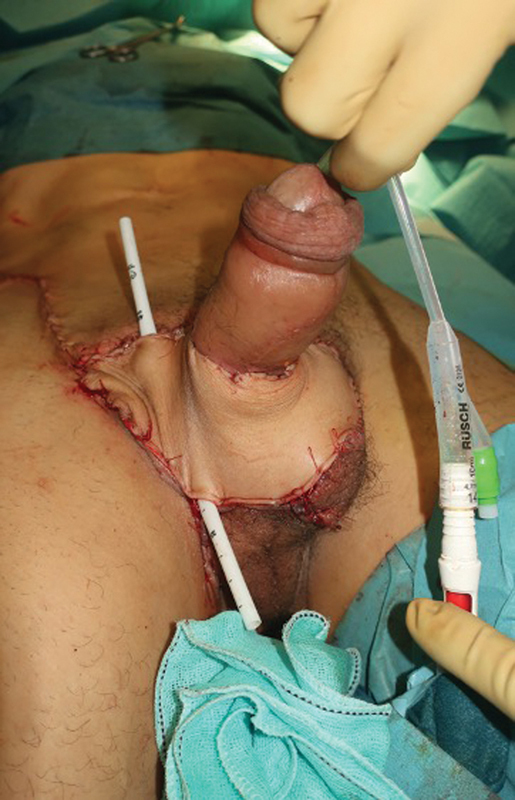
Immediate postoperative image after reconstruction of the penoscrotal defect with the SCIP flap. SCIP, superficial circumflex iliac artery perforator.

Subsequently, an SCIP flap measuring 20 cm in length and 8 cm in width was meticulously designed in an oblique–vertical orientation, positioned cranial and medial to the ASIS using the superficial branch of the right SCIA as source vessel and pivot point ( Figs. 4 and 5 ). The vascular flap pattern was axial type. 15 Once the superficial branch of the SCIA was identified and isolated, the flap was carefully harvested from distal to proximal along the oblique muscle fascia and rotated 160 degree clockwise to reach the site of the defect ( Figs. 6 and 7 ). The viability of the flap was assessed through clinical evaluation and indocyanine green angiography. To address the remaining small dorsal penis defect, a split-thickness skin graft was employed.

**Fig. 8:**
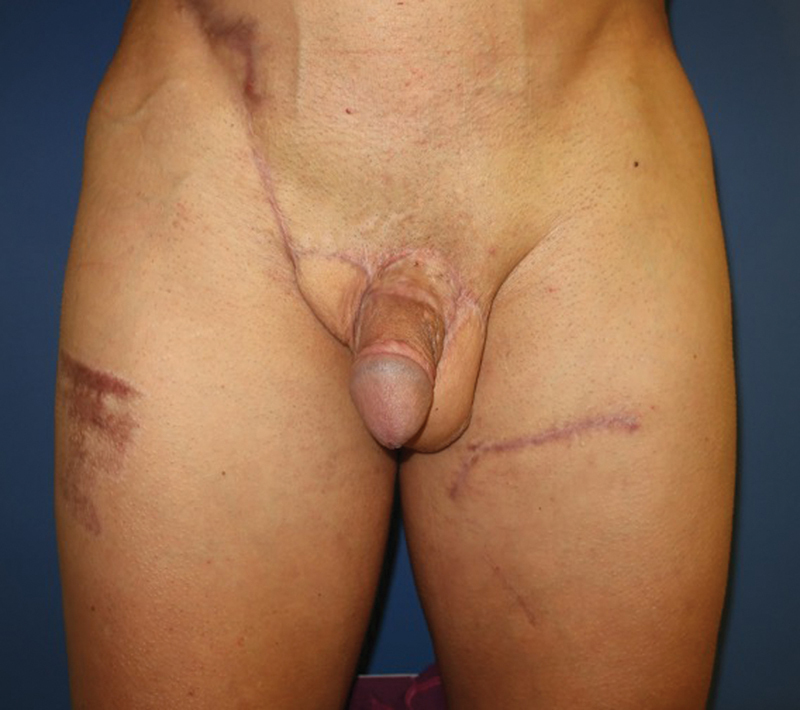
Six months after surgery.

During the postoperative 6-month follow-up evaluation, a satisfactory outcome was observed, characterized by acceptable cosmetic results and the absence of lymphorrhea or wound dehiscence ( Fig. 8 ). Notably, normal testicular function was maintained following the surgery, as evidenced by normal blood-free testosterone levels.

## Discussion

Penoscrotal skin is loose, ample, elastic, expandable, and thinner than in other places, with a thin subcutaneous layer. Proper functional and aesthetic reconstruction of the penoscrotal tissue is important for patient confidence, especially after a traumatic injury, as it usually happens in younger patients. Therefore, it is crucial to prioritize the restoration of the original form of the penis or scrotum while ensuring the preservation of function. In cases where small defects are present, a scrotal flap may be enough for coverage. However, for larger defects where there is insufficient scrotal tissue, alternative techniques should be employed for reconstruction. While split-thickness skin grafts offer a relatively straightforward approach, they lack the stability of flaps, and the potential for scar contracture can result in the loss of the normal cremaster muscle reflex. 9 On the other hand, the use of local or free flaps that are thick and bulky presents a potential concern as they can elevate testicular temperature, which in turn may impact both spermatogenesis and cosmesis.

In contrast to the previously mentioned approaches, the local SCIP flap offers numerous benefits. It serves as a reliable flap that is naturally thin, situated in close proximity to the defect site, and facilitates a relatively straightforward tissue transfer. 1 Furthermore, using a local pedicled SCIP flap, instead of a free flap, avoids the requirement of microsurgical anastomosis or recipient vessels dissection, shortening surgical time. Limited reports exist regarding the utilization of the SCIP flap in either propeller or pedicled form for penoscrotal reconstruction. 1 11 12 16

We define the flap used in this case as the “extended” or “direct” SCIP flap, and it differs from the conventional SCIP flap in several ways. Our variation of SCIP flap is exclusively based on the superficial branch of the SCIA, which offers a consistent and lengthy vascular pedicle with an axial pattern. The skin paddle of our flap is based on the abdominal skin positioned in a medial and cranial location relative to the ASIS. 14 Therefore, it differs from the “classic” SCIP flap described by Koshima, which relies on the deep branch perforators and features a skin paddle situated laterally and proximally to the ASIS. 10 Furthermore, our approach also distinguishes itself from previously reported variations of the SCIP flap. 17 18 19 20 21

It also differs from the proposal made by J.P. Hong. 22 This last one is a pure perforator flap with short pedicle (based either on a cutaneous perforator from the superficial or deep branch) that needs perforator-to-perforator anastomosis in free designs. It is dissected in a suprafascial plane (above the superficialis fascia) with a different cutaneous territory (caudal and medial to the ASIS, between this and the pubis). In contrast, the cutaneous territory of our flap can be displaced proximally to obtain a larger flap because we always harvest it on the superficial branch of the SCIA, which has a constant presence and follows an axial pattern proximal and medial to the ASIS until the 12th rib. We also perform, if needed, a complete dissection of the SCIA until its origin in the common femoral artery, which provides us with a longer and larger pedicle for a safer flap mobilization.

Herein, the “extended” SCIP flap variation offers some additional advantages to conventional SCIP flap: larger flaps with direct closure of the donor area, it avoids injury over deep lymphatic nodes (preventing lymphedema development), and it provides thin and more pliable elastic skin. The pedicle is longer, with larger caliber of the vessels, providing a wider and safer pivot point. Also, this surgical method avoids the requirement of supramycrosurgical techniques for flap anastomosis in free designs.

In contrast with other publications, 15 in the anatomical and clinical study that we recently performed, 14 we found the “extended” SCIP flap pedicle, based on the superficial branch of the SCIA, to be anatomically constant. The skin paddle design that we describe, also differs from other SCIP flap variations previously reported. 1 17 20 As we mentioned before, our flap uses the abdominal skin situated medial and cranial to the ASIS and is based exclusively on the superficial branch of the SCIA.

Although it is a very good option for thin people, in obese patients, the “extended” SCIP flap can be too thick for the ideal reconstruction of the penoscrotal area. This is why we recommend to always check it, prior to surgery, and look for other flap options if necessary.

We also propose the use of the “extended” SCIP flap variation, in a local or free design, as an ideal flap for the repair of complex three-dimensional tissue defects where thin flaps with medium or large dimensions are required. Some of this areas are the leg, foot, forearm, hand, head and neck and penoscrotal area.

In conclusion, the use of the “extended” or “direct” pedicled SCIP flap for penoscrotal reconstruction following a traumatic injury proves to be a highly effective approach, particularly due to its compatibility with the unique characteristics of penoscrotal tissue. However, like other pedicled flaps, it is crucial to exercise caution during the procedure to avoid complications such as pedicle kinking.
